# Estimating lead time and overdiagnosis in cancer screening programmes: the MOCCI method

**DOI:** 10.1093/ije/dyag167

**Published:** 2026-08-19

**Authors:** Bor Vratanar, Maja Pohar Perme

**Affiliations:** Institute for Biostatistics and Medical Informatics, University of Ljubljana, Ljubljana, Slovenia; Institute for Biostatistics and Medical Informatics, University of Ljubljana, Ljubljana, Slovenia

**Keywords:** lead time, overdiagnosis, counterfactual, deconvolution, causal inference, incidence

## Abstract

**Background:**

Cancer screening enables earlier cancer diagnosis and treatment. The interval between cancer diagnosis by screening and the symptomatic detection in the absence of screening is termed lead time. If a patient’s tumour, detected by screening, would never surface clinically, the patient is considered overdiagnosed. Estimating these quantities is crucial for evaluating cancer screening programmes.

**Development:**

We developed MOCCI (Minimizing Observed and Counterfactual Cancer Incidence), a novel parametric method for estimating the lead time distribution. MOCCI compares age- and calendar-stratified cancer incidence between individuals invited to screening and those not invited and estimates the lead time distribution that minimizes the incidence difference between the two groups. The probability of overdiagnosis is then estimated by comparing the model-predicted lead time with the time to death from other causes.

**Application:**

In an application to the Slovenian breast cancer screening programme, we estimated that 30% (95% confidence interval [CI], 20% to 39%) of screen-detected cases were non-progressive and 33% (95% CI, 25% to 43%) were overdiagnosed; assuming an exponential lead time distribution for progressive cancers, the mean lead time was 1.8 years (95% CI, 1.3 to 2.9).

**Conclusions:**

This study proposes a new method for estimating lead time and overdiagnosis. The proposed method (a) can accommodate various lead time distributions, (b) yields a lead time distribution that is aligned with the observed excess incidence arising from screening, and (c) enables the separation of different sources of overdiagnosis. The method provides stable estimates when sample sizes are large and the assumed lead time model is simple.

Key Messages

- We present a new parametric method for estimating the lead time distribution and the probability of overdiagnosis in cancer screening programmes.
- The proposed method provided a good fit to real-world data; when applied to the Slovenian breast cancer screening programme, the estimated probability of overdiagnosis was 33% (95% confidence interval [CI], 25% to 43%), and the mean lead time among progressive screen-detected cases was 1.8 years (95% CI, 1.3 to 2.9).
- The method enables separate estimation of overdiagnosis arising from non-progressive cancers and from progressive cancers that would not otherwise have been clinically diagnosed during a person’s lifetime.

## Introduction

In cancer screening programmes, participants are screened every few years with medical tests to detect and treat cancer early, before symptoms appear. Individuals invited to participate in screening may remain cancer-free (*no cancer cases*), have their cancer detected by a screening test (*screen-detected cases*), or be diagnosed with cancer based on symptoms (*symptomatic cases*). Symptomatic cases include cancers diagnosed between scheduled screenings (interval cancers) or among individuals who do not attend the screening programme.

*Lead time* is commonly defined as the interval between a screen diagnosis and the hypothetical symptomatic (clinical) detection of cancer in the absence of screening. If lead time exceeds the time from screen diagnosis until death, the individual is considered to be *overdiagnosed* [1–3].

In this study, we focus on estimating the distributions of both lead time and overdiagnosis. Accurate knowledge of these quantities is essential for several reasons: (a) to obtain bias-corrected survival estimates for screen-detected cancers [4], (b) to inform policy and patient decisions about the risk of overtreatment [5], and (c) to calibrate simulation models (e.g. Forastero *et al.* [6] and Koleva-Kolarova *et al.* [7]) so that simulated data align with reality. As part of the programme evaluation, we aim to estimate lead time and overdiagnosis in the Slovenian breast cancer screening programme [8].

Historically, to estimate lead time, research has focused on estimating the mean sojourn time, defined as the period during which a tumour remains asymptomatic yet detectable by screening [3]. Two main approaches have been used (for a review, see Cheung *et al.* [9]): recurrence time models and Markov chain models. Typically, sojourn time is assumed to follow an exponential distribution, which allows for the derivation of lead time [3, 10]. More recently, biological tumour-growth models (specific to breast cancer screening programmes) have been introduced. These models jointly model the unobservable processes of tumour growth, screening sensitivity, and symptomatic detection [11–14]. The resulting parameters can be used to predict lead time at the individual level [15].

The probability of overdiagnosis can be estimated in two ways [16–18]. The *excess incidence approach* uses the difference in overall incidence, referred to as excess incidence, between invited and non-invited groups as a proxy for overdiagnosis. This approach may be biased when follow-up is limited: increases in incidence may reflect not only overdiagnosed cases but also cancers detected earlier by screening due to lead time, leading to overestimation. However, with sufficiently long follow-up, this bias is expected to diminish [17]. The second approach, the *lead time approach*, estimates the probability of overdiagnosis by comparing model-predicted lead time with the time to death from other causes [17]. If the model and its assumptions are valid, this method should yield unbiased estimates.

Previous studies have consistently reported substantial discrepancies between overdiagnosis estimates obtained from the two approaches [16, 19]. Typically, the excess incidence approach is preferred because it is more directly grounded in observed data [20]. The large differences that arise with the lead time approach (relative to the excess incidence approach) are attributed to model misspecification or unrealistic assumptions of lead time models [20]. These discrepancies do not invalidate the lead time approach per se [18], but they highlight the need for continued work to assess and improve lead time models.

In this study, we propose MOCCI (Minimizing Observed and Counterfactual Cancer Incidence; see the Estimator section), a novel approach for estimating lead time and overdiagnosis, applicable to screening programmes designed to detect and treat early-stage cancers (e.g. breast cancer). Compared with previous approaches, MOCCI (a) may accommodate various lead time distributions, including mixture models that allow a fraction of cancers to be non-progressive, (b) yields a lead time distribution that is aligned with the observed excess incidence arising from screening, and (c) allows separate estimation of the proportions of overdiagnosis attributable to non-progressive cancers and to competing mortality in progressive cancers. We use the term ‘non-progressive’ to include both truly non-progressive and regressive preclinical cancers.

## MOCCI

### Input data

The MOCCI method is designed for settings in which cancer incidence can be compared between individuals invited to a newly implemented screening programme and individuals not invited. Such data may arise from (a) randomized controlled trials, (b) natural experiments (i.e. difference-in-difference studies [21]), or (c) studies comparing concurrent and historical groups. Depending on the study design, the data may come from one or several separate sources. The MOCCI method requires the following:

1. Cancer patient data, including age and date of diagnosis, invitation status (invited or not invited to screening), relevant demographic variables (e.g., sex, race), mode of detection (screen-detected or symptomatic), and date of last follow-up.
2. Person-years at risk, stratified by date (partitioned into calendar years), age (partitioned into 1-year intervals), and invitation status.
3. Data on general population mortality (life tables), stratified by date (partitioned into calendar years), age (partitioned into 1-year intervals), and relevant demographic variables.

### Thought experiment

**Figure 1:**
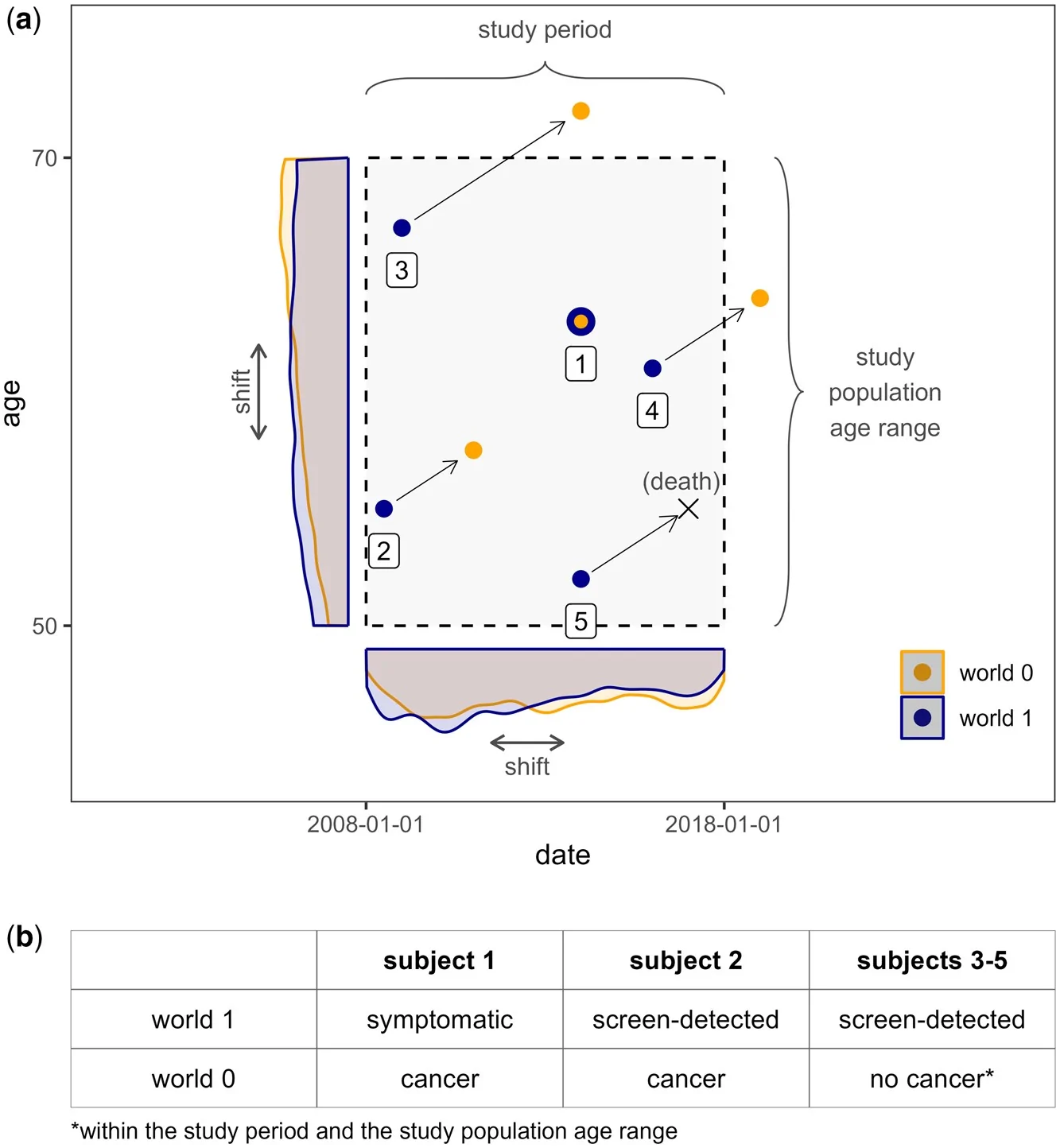
(a) Lexis diagram for subjects 1–5 (see example in text). Points mark cancer diagnosis in world 0 or world 1; arrows connect the same individual. Subject 5 dies from other causes before a cancer diagnosis occurs in world 0. Side plots illustrate possible marginal distributions of calendar date and age at cancer diagnosis in each world. (b) Observed detection mode in world 0 and in world 1.

To understand the basis of the MOCCI method, we begin with a thought experiment. Imagine a breast cancer screening programme launched in 2008 that invites all women aged 50 to 70 years in a given country to participate in screening every 2 years. The study concludes in 2018. During this period, we observe some cancers being diagnosed by screening and others based on symptoms. For clarity, we restrict attention to cancers diagnosed between ages 50 and 70 (the study population age range). As an illustration, we select five cancer cases from our thought experiment, shown as blue points in Fig. 1a. Subject 1 is diagnosed based on symptoms, while subjects 2–5 are screen-detected. We then ask:

*What would have happened if the screening programme had never been implemented?*

In the context of the counterfactual framework introduced in our previous work [4], we refer to the scenario with the screening programme as world 1 (presence of screening) and to the (counterfactual) scenario without it as world 0 (absence of screening). In Fig. 1a, orange points denote cancer diagnoses that would occur in world 0; arrows connect the same individual. By comparing data from world 1 with data from world 0, we observe the following:

- Symptomatic cases (subject 1) in world 1 are assumed to be diagnosed at the same time as in world 0 (blue points overlap with orange points).
- Screen-detected cases (subjects 2–5) in world 1 are assumed to be diagnosed earlier than in world 0 due to lead time (i.e. lead time is assumed to be positive).
- Within our study period and the study population age range, some cancers occur only in world 1 and not in world 0 (subjects 3–5). This happens because individuals might be diagnosed with cancer outside the study population age range (subject 3), be subject to administrative censoring (subject 4), or die from other causes before a diagnosis in world 0 (subject 5). Collectively, we refer to these as *excess* cases. Note that the upper limit for the study population age range is arbitrary and could be extended; doing so could reclassify subject 3 as non-excess.

The five subjects were selected because they represent all possible unique combinations of events in world 0 and world 1 (Fig. 1b).

At the population level, lead time induces a *temporal shift* in the joint distribution of the date and age of diagnosis between world 0 and world 1; for simplicity, only the corresponding shifts in the marginal distributions are shown in Fig. 1a (i.e. the side plots). In addition, because of the excess cases (which arise due to lead time), cancer incidence is higher in world 1 than in world 0.

### Estimator

We now turn to real-world data and explain the proposed estimator of lead time.

Assume for a moment that the data come from a randomized controlled trial in which the screening programme was introduced for the first time. Since we cannot observe each individual in both worlds, we rely on two separate data sources: individuals invited to screening (corresponding to world 1) and those not invited (corresponding to world 0). Based on the thought experiment, we expect the two datasets to differ in two respects: (1) there will be a temporal shift in the date and age of cancer diagnosis, and (2) the invited group will have a higher cancer incidence. The core idea of the MOCCI method is to use both pieces of information to estimate (reconstruct) lead time.

**Figure 2:**
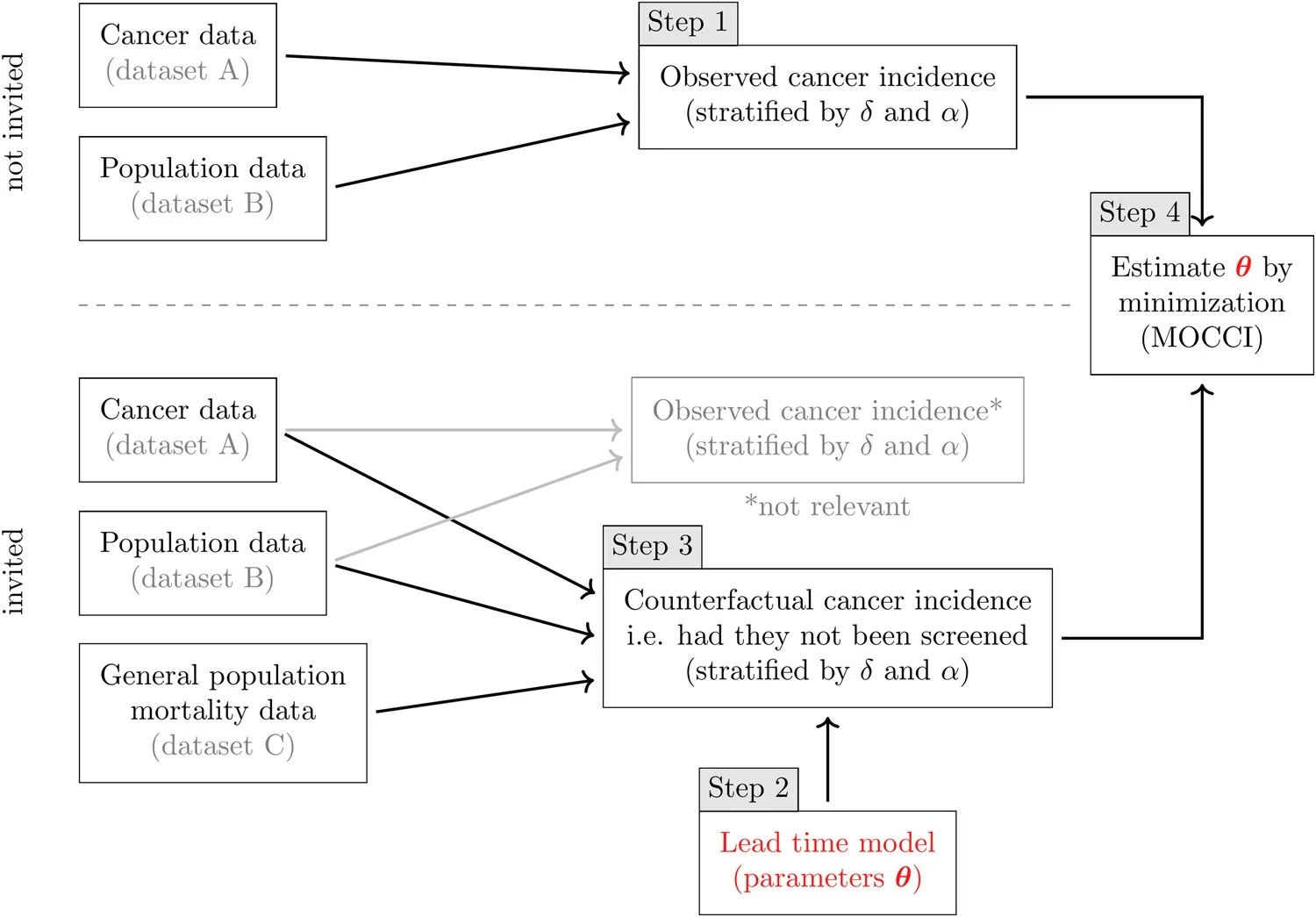
Overview of the MOCCI method (Minimizing the Observed and Counterfactual Cancer Incidence); δ denotes calendar year, and α denotes 1-year age intervals. Explanations of each step are provided in the text.

The MOCCI method proceeds as follows (see also Fig. 2):

1. Observed cancer incidence. First, for the non-invited group, we partition the date and age of cancer diagnosis into 1-year intervals, forming a grid of 1 × 1-year cells indexed by calendar year δ and age interval α. Within each cell (δ, α), we calculate cancer incidence in the non-invited group using the following formula:
  $$\begin{array}{c} \text{Incidence}\;(\delta ,\alpha )=\frac{\text{Number}\;\text{of}\;\text{cancer}\;\text{cases}\;\text{in}\;(\delta ,\alpha )}{\text{Person-years}\;\text{at}\;\text{risk}\;\text{in}\;(\delta ,\alpha )} \end{array}$$
2. Lead time distribution. Second, we assume that the lead time $L\;\mathrm{for}\;\mathrm{screen}-\mathrm{detected}\;\mathrm{cases}\;(\mathrm{in}\;\mathrm{the}\;\mathrm{invited}\;\mathrm{group})\;\mathrm{follows}\;a\;\mathrm{parametric}\;\mathrm{distribution}\;\mathrm{with}\;\mathrm{density}\;f_{L}(l|\theta ),\;\mathrm{where}\;\theta$ denotes the model parameters to be estimated. This distribution may take any parametric form and may include a point mass at infinity to represent non-progressive tumours.
3. Counterfactual cancer incidence. Next, conditional on the parameters θ, we estimate the counterfactual cancer incidence in the invited group (i.e. the incidence we would observe if this group had not been invited). These estimates account for both other-cause mortality and censoring.
4. Likelihood. Lastly, we estimate the parameters θ by minimizing the discrepancy (shift) between observed (step 1) and counterfactual (step 3) cancer incidence across all cells in the grid, using a maximum likelihood approach.

Once the parameters θ are estimated, the probability of overdiagnosis can be estimated directly via the lead time approach [17].

Since the method estimates lead time by Minimizing the Observed and Counterfactual Cancer Incidence, we refer to it as the MOCCI method. A formal derivation of the MOCCI method is provided in the supplementary material, Section 1. The results from the simulation study in the supplementary material, Section 2: (a) illustrate the consistency of the estimator, (b) show that the method can reliably distinguish between data-generating mechanisms in which the proportion of non-progressive cancers is zero and those in which it is non-zero, and (c) indicate that large samples, typically a few thousand cases in each group, are needed to obtain stable estimates.

### Assumptions

For data obtained from a randomized clinical trial, the MOCCI estimator relies on the following assumptions:

- The parametric form of the lead time distribution for screen-detected cases is correctly specified.
- Lead time distribution is the same for all screen-detected cancers
- Lead time for symptomatic cases is zero.
- The hazard of other-cause (competing) mortality is identical in both groups and does not depend on the timing of treatment.

Additional assumptions depend on the study design and data source. For example, when applying MOCCI to studies comparing concurrent and historical cohorts, one must use pre-screening data to project incidence rates into the screening era; this requires additional assumptions about how background incidence changes over time. When applying MOCCI to natural experiment, one must assume that the groups would have had similar cancer incidence in the absence of screening, or otherwise account for any imbalance between them.

## Empirical example

### Study design

We applied the MOCCI method to data from the Slovenian breast cancer screening programme. The programme began in 2008 and invites women aged 50–69 to attend mammography screening every 2 years. Initially limited to the central region of the country, it was gradually expanded to cover all regions by the beginning of 2019. This rollout created a natural experiment in which some women were invited to participate in screening while others were not. In this study, we assume that, in the absence of screening, the age-specific cancer incidence would be the same in the invited and non-invited groups. The effectiveness of the programme has already been evaluated in previous studies [8, 22, 23].

### Data

In the Input data section, we described the three datasets required for the MOCCI method. In our case, each dataset was obtained as follows:

1. Data on breast cancer patients, including ductal carcinoma *in situ* (DCIS) cases, were obtained from the Slovenian cancer registry [24] and the Slovenian breast cancer screening registry [8], then matched using unique identifiers. The analysis was restricted to women aged 50–75 years at the time of diagnosis. The non-invited group comprises 10 801 (7.3% DCIS cases), diagnosed in women either between 2002 and 2008 (before the programme began) or, for women not yet invited, between 2008 and 2019. The invited group includes 4285 (15.6% DCIS cases) breast cancer cases diagnosed from 2008 to 2020, of which 2894 were screen-detected (20.6% DCIS cases).
2. Population counts of Slovenian women at risk, stratified by δ and α, were obtained from the Statistical Office of the Republic of Slovenia covering the entire population. Person-years at risk for the invited group, stratified by δ and α, were calculated from the screening registry. Person-years for the non-invited group were derived by subtracting these from the total. Because the screening programme invites only women without a prior cancer diagnosis, we adjusted the population at risk in the invited group to also account for women with a previous cancer; see supplementary material, Section 3.1, for details.
3. Mortality data for the Slovenian general population, stratified by δ, α, and sex, were obtained from the Statistical Office of the Republic of Slovenia.

### Statistical analysis

The MOCCI method was used to estimate lead time and the probability of overdiagnosis. An age–period–cohort model [25] was fitted to cancer incidence in the non-invited group (step 1 in the Estimator section), including pre-programme cases diagnosed between 2002 and 2008 to improve the stability of the estimated underlying incidence. This also allowed for prediction beyond the training period, during 2019–20. To predict counterfactual cancer incidence in the invited group, given an assumed lead time distribution (steps 2 and 3 in the Estimator section), we considered two models. In model A, all screen-detected cancers were assumed to be progressive, and the lead time was assumed to follow exponential distribution. In model B, a fraction of screen-detected cancers was assumed to be non-progressive, while the lead time for progressive cancers followed an exponential distribution (i.e. a mixture model). Model A is nested within model B as the special case when the fraction of non-progressive cancers is set to 0. The parameters were estimated by numerically maximizing the likelihood described in the Estimator section, and their standard errors were obtained from the inverse of the observed Fisher information matrix. Model fit was evaluated using the Poisson deviance, calculated from the discrepancies between observed and model-predicted cancer counts in each age–year cell [26]. The two nested models were compared based on the difference in deviance. The probability of overdiagnosis was estimated using both the lead time approach (see supplementary material, Section 1, for details) and the excess incidence approach [17].

The analysis was conducted in the R programming language [27]. The complete workflow is provided in supplementary material, Section 3.2.

### Results

**Figure 3:**
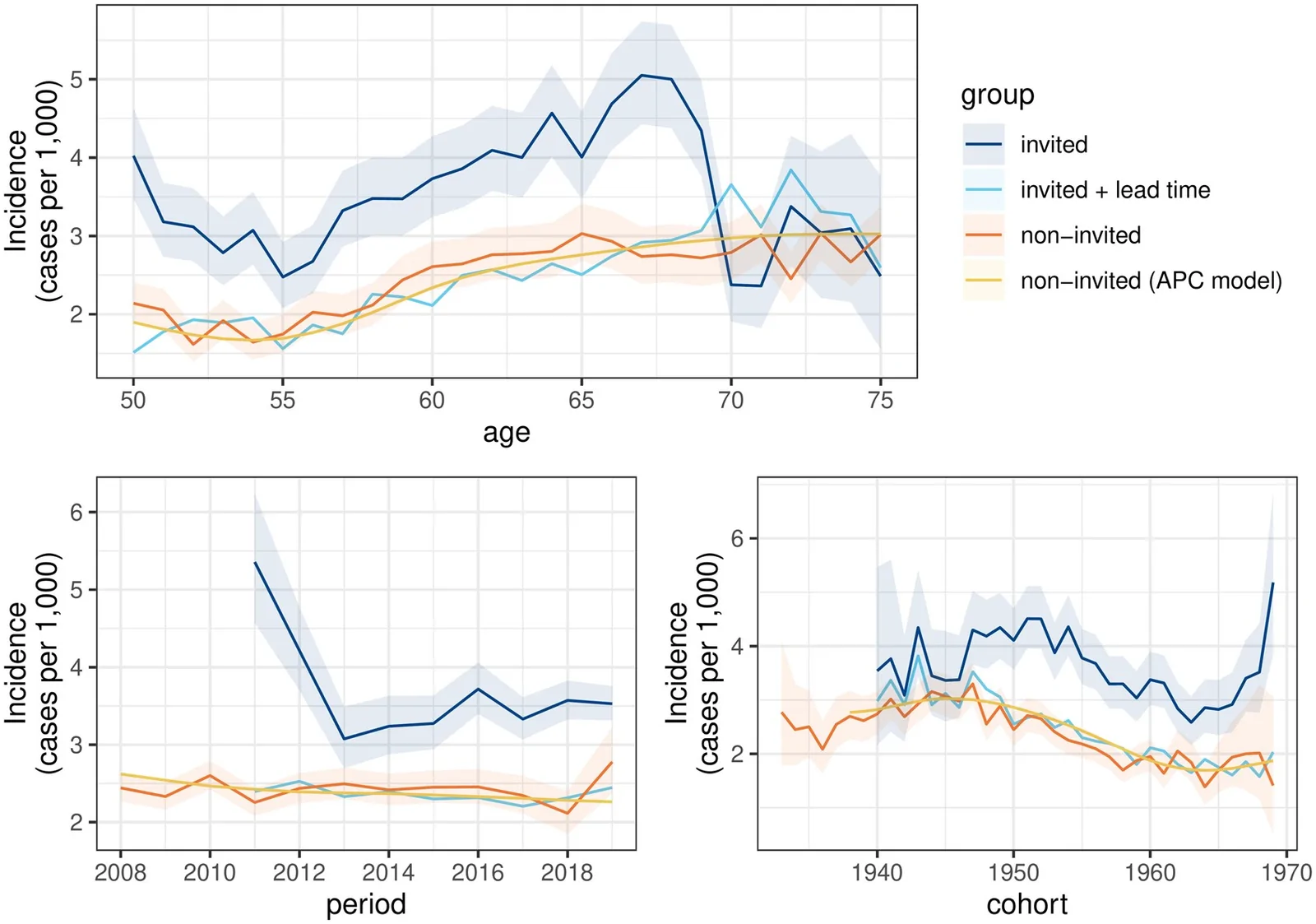
Breast cancer incidence by age, period, and birth cohort for invited and non-invited groups. Shaded area corresponds to 95% confidence intervals. Incidence before 2011 and for cohorts born before 1940 was omitted from the display because of small sample sizes (and consequently high variance). APC model = age–period–cohort model; ‘invited + lead time’ represents back-estimated incidence obtained using the estimated lead time parameters from model B.

In Fig. 3, we first show cancer incidence in the invited and non-invited groups by calendar period, age, and birth cohort. Incidence is consistently higher in the invited group, with a ‘compensatory drop’ [16] after age 70, when screening ceases.

We then fitted both model A and model B to the data; the results are shown in Table 1. The deviance was lower for model B than for model A (Δ deviance = 12.3, df = 1, P<.001), indicating that model B provided a better fit. Under model A, the estimated rate was 0.26, corresponding to a mean lead time of 3.8 years. This relatively large mean lead time reflects the model’s attempt to compensate for excess incidence. In contrast, model B achieves a better fit by allowing a proportion of screen-detected cancers to be non-progressive (i.e. to have infinite lead time). The estimates from model B indicate that the rate of the exponential lead time distribution for progressive screen-detected cancers is 0.56, corresponding to a mean lead time of 1.8 years, and that 30% of all screen-detected cancers were assigned to the non-progressive component of the model. Given its superior fit to the data, we retained model B. The fit of model B in absolute terms was then evaluated by back-estimating the counterfactual no-screening incidence for the invited group and comparing it with the observed incidence in the non-invited group; the two estimates agree reasonably well (see Fig. 3).

**Table 1:** Parameter estimates [95% confidence intervals] and model fit statistics for models A and B

Model	Rate (progressive)	Prob. (non-progressive)	Deviance	df	Deviance/df
A	0.26 [0.24–0.28]	Fixed to 0	214.3	296	0.72
B	0.56 [0.35–0.76]	0.30 [0.20–0.39]	202.0	295	0.68

df were calculated as the number of age-year cells where the size of the population at risk was larger than 0.

**Figure 4:**
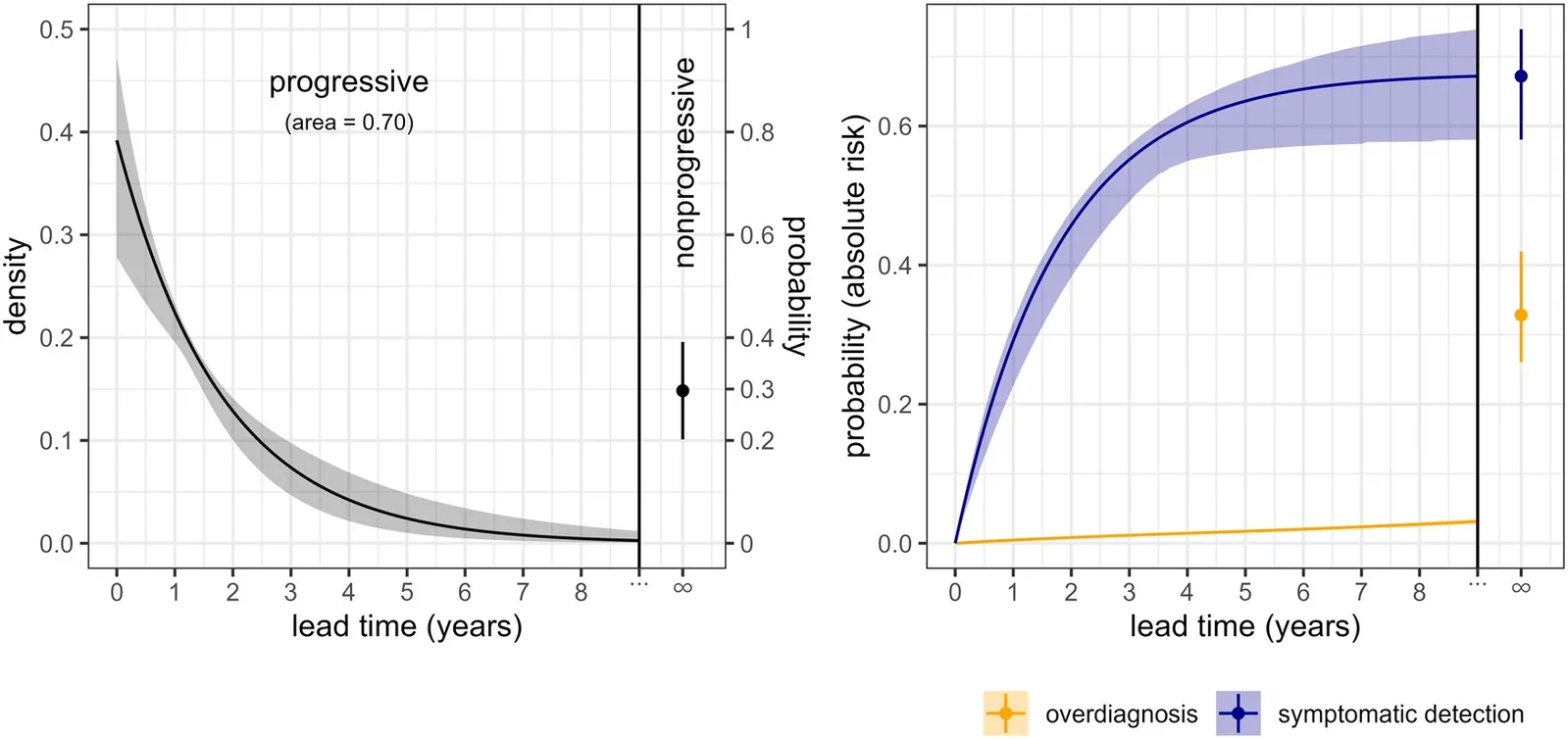
Left: The estimated density of lead time for progressive cancers (primary y-axis) and the proportion of non-progressive cancers as time approaches infinity (secondary y-axis). Shaded area corresponds to 95% confidence intervals. Right: The absolute risk for overdiagnosis and symptomatic detection. Shaded area corresponds to 95% confidence intervals.

Figure 4 (left) shows the estimated density of lead time (based on model B) for screen-detected cases. Since MOCCI targets the *latent* lead time distribution, the estimand is the distribution that would be observed in the absence of other-cause mortality and censoring. To understand how lead time interacts with other-cause mortality, we model it in a competing risk setting. Each cancer that would be screen-detected in world 1 is subject to two competing events in world 0, from the time of hypothetical screen diagnosis onward: death from other causes (i.e. overdiagnosis) or symptomatic diagnosis. Figure 4 (right) shows the absolute risk for each event. We have estimated that as time tends to infinity, the overall probability of overdiagnosis approaches 33%. In comparison, using the excess incidence approach, we estimated that 36% of screen-detected cancers were overdiagnosed. The decomposition of overdiagnosis is straightforward: 30% of all screen-detected cancers were estimated to be non-progressive and are therefore, by definition, overdiagnosed; the remaining 3% must therefore represent progressive cancers that are also overdiagnosed.

## Discussion

Cancer screening leads to earlier diagnosis, shifting cancer diagnoses earlier in both age and calendar time, while increasing overall incidence. In this paper, we propose a new method that exploits this information to estimate lead time.

Existing approaches to modelling lead time have often been viewed with scepticism because the implied overdiagnosis derived from the estimated lead time models has differed substantially from the excess incidence observed between invited and non-invited groups [16, 19]. The proposed MOCCI method closes this long-standing gap in the literature by directly incorporating the excess incidence information into the estimation process. This ensures that the resulting lead time distribution is aligned with differences in overall cancer incidence between invited and non-invited groups.

Like all methods, MOCCI relies on several assumptions, which we have tried to make explicit in this work (see the Assumptions section). Equally important, however, is what MOCCI does not assume: in particular, it does not require complete adherence to the screening programme, does not assume that all cancers are progressive, and does not assume cancer incidence to be stable over calendar time. Assumptions of progressive disease and stable cancer incidence are common in existing methods (e.g. Zelen and Feinleib [3], Isheden and Humphreys [12], and Shen [28]).

A practical limitation of MOCCI is identifiability: in our experience, the method can reliably recover parameters only for simple lead time models, roughly of the order of two parameters. Thus, although one could in principle allow lead time to have a more complex shape or to depend on stage, subtype, or other covariates, such extensions are unlikely to be estimable in a stable way from the available data.

Further work should evaluate MOCCI across different screening settings and lead time distributions, and compare its performance with existing estimators. Extensions to programmes in which screening can prevent invasive cancers (e.g. cervical screening) would be of interest. Combining MOCCI with biological tumour growth models [13] may also allow tumour size information to inform estimation.

## Ethics approval

Ethical approval and individual informed consent were not required for this study because it involved the secondary analysis of routinely collected, pseudonymized registry data under the applicable institutional and national regulations.

## Supplementary Material

dyag167_Supplementary_Data

## Data Availability

The individual-level data underlying this article cannot be shared publicly because they contain confidential health information and were provided subject to data-use restrictions.
