# Supplementary material for: Estimating lead time and overdiagnosis in cancer screening programmes: the MOCCI method
Source: Int J Epidemiol. 2026 Aug 19;55(5):dyag167. doi: 10.1093/ije/dyag167 (PMC13487635; doi:10.1093/ije/dyag167)
Supplement: Supplementary file 1 [file dyag167_supplementary_data.pdf]

Supplementary material

Estimating lead time and overdiagnosis in cancer screening programmes: the MOCCI method

Bor Vratanar and Maja Pohar Perme

Table of contents

1 Formal derivation of the MOCCI method **2**
1.1 General notation . . . 2
1.1.1 Potential outcomes . . . 2
1.1.2 Study period and eligible age range . . . 2
1.1.3 Observed random variables . . . 2
1.1.4 Identifying conditions . . . 3
1.1.5 Hypothetical random variables . . . 3
1.2 Estimator derivation . . . 3
1.2.1 Lead time distribution . . . 3
1.2.2 Observed cancer incidence . . . 3
1.2.3 Counterfactual cancer incidence . . . 4
1.2.4 Likelihood . . . 6
1.2.5 Relaxing assumptions . . . 7
1.3 Estimating the probability of overdiagnosis . . . 8

2 Simulation study **9**
2.1 Aims . . . 9
2.2 Setup . . . 9
2.2.1 Data generating mechanism (DGM) . . . 9
2.2.2 Estimands . . . 10
2.2.3 Methods . . . 10
2.2.4 Performance measures . . . 10
2.2.5 Hardware, software and computing time . . . 11
2.3 Results . . . 11
2.3.1 Properties of the MOCCI method . . . 11
2.3.2 Accuracy in the presence of non-progressive cancers . . . 11

3 Empirical example **13**
3.1 Person-years at risk calculations . . . 13
3.2 Complete workflow . . . 13

Section 1: Formal derivation of the MOCCI method

1.1 General notation

1.1.1 Potential outcomes

The intervention of interest is the invitation to the screening programme, denoted by X, where $X = 0$ represents the control group (not invited) and $X = 1$ represents the experimental group (invited). The main event of interest is cancer diagnosis, which may occur at different times depending on whether an individual is invited to the CSP. To formalize this, we adopt the notation of potential outcomes [2, 3]. For any outcome Y, let $Y^{(0)}$ be the potential outcome if the participant is not invited to the programme (world 0), and $Y^{(1)}$ be the potential outcome if the participant is invited (world 1). In reality, we can observe each participant in only one of the two worlds. We assume consistency, meaning the observed outcome matches the potential outcome corresponding to the actual assignment.

1.1.2 Study period and eligible age range

The study spans several consecutive years, from $\delta_{\min}$, marking the *start* of the screening programme, to $\delta_{\max}$, at which point the study ends. It includes participants aged $\alpha_{\min}$ to $\alpha_{\max}$, representing the lower and upper screening ages, respectively. New subjects may be invited to participate in the study each year. Let n_x denote the total sample size in group $X = x$ (including no cancer cases), for $x \in \{0, 1\}$. Within each group, index participants by $i \in \{1, 2, \ldots, n_x\}$.

1.1.3 Observed random variables

The primary event of interest is cancer diagnosis. Let A represent the age at cancer diagnosis and D the date of diagnosis. Participants may be censored or die due to competing risks before a cancer diagnosis can be made. The age at competing risk or censoring is indicated by C with a subscript to denote different possibilities:

- C_1: Age at death due to other causes. Assumed to be the same in world 0 and world 1.
- C_2: Age at the end of the study. Given an individual's date of birth and a fixed study end date ($\delta_{\max}$), C_2 is deterministic and thus identical in world 0 and world 1.
- C_3: Age at which the individual exits our target population. For simplicity, we set this age to $\alpha_{\max}$ for the moment. This quantity is fixed and identical in world 0 and world 1. In practice, the exit age may be chosen to exceed $\alpha_{\max}$; this will allow more data to enter the estimation process introduced later.

The age C, is the minimum of these, expressed as $C = \min(C_1, C_2, C_3)$. Cancer cases are defined by $A < C$.

At the end of the study, each participant can be classified into one of three groups based on the mode of cancer detection, denoted by M:

- No cancer ($M = noc$), $A \geq C$
- Symptomatic ($M = symp$): cancer diagnosed based on symptoms
- Screen-detected ($M = scr$): cancer diagnosed based on screening test

Note that our definition of symptomatic cancers in world 1 includes interval cancers as well as cancer cases that do not attend screening programmes. In world 0, all cancer cases are symptomatic since no screening takes place.

1.1.4 Identifying conditions

To define lead time and overdiagnosis in our counterfactual framework, we require the following identifying conditions (i.e. assumptions required to define the estimands of interest):

- In both worlds, participants die due to other causes at the same age, i.e., $C_1^{(0)} = C_1^{(1)}$. This also implies that $C^{(0)} = C^{(1)}$.
- The age at diagnosis for cancers detected based on symptoms in world 1 is assumed to be the same as in world 0, i.e., $A^{(0)} = A^{(1)}$ if $M^{(1)} = symp$.
- For screen-detected cancers, the diagnosis occurs at an earlier time in world 1 compared to world 0, i.e. $A^{(1)} < A^{(0)}$ if $M^{(1)} = scr$.
- Death due to cancer can occur in either world, but only after the time at which a symptomatic diagnosis would occur in world 0.

We assume conditions above hold throughout this work.

1.1.5 Hypothetical random variables

Lead time and overdiagnosis can only be defined by considering their values in both world 0 and in world 1. As they do not inherently belong to either world, we omit the world-specific notation for them.

Lead time (L) is defined as a difference in age at cancer diagnosis between the two worlds:

$$L = \begin{cases} A^{(0)} - A^{(1)} & ; \quad M^{(1)} = scr \\ 0 & ; \quad M^{(1)} = symp \\ \text{not defined} & ; \quad M^{(1)} = noc \end{cases}$$

Overdiagnosis (O) is a binary indicator that takes the value 1 if a cancer is overdiagnosed (i.e., if death due to other causes occurs between the cancer diagnoses in world 1 and world 0) and 0 otherwise:

$$O = \begin{cases} I(A^{(1)} < C_1^{(0)} \leq A^{(0)}) & ; \quad M^{(1)} = scr \\ 0 & ; \quad M^{(1)} = symp \\ \text{not defined} & ; \quad M^{(1)} = noc \end{cases}$$

Note that for symptomatic cancers, $L = 0$ and $O = 0$, since their age at cancer diagnosis is assumed to be the same in both worlds.

1.2 Estimator derivation

1.2.1 Lead time distribution

The lead time for all cancer cases follows a mixture distribution: symptomatic cancers have a lead time of 0, while screen-detected cancers follow the density $f_L(l)$, defined for $l \geq 0$. We focus on $f_L(l)$, which may take any parametric form, including a mixture model with a proportion of non-progressive cancers, assumed to have infinite lead time values. In our model, this is parameterized as $f_L(l \mid \boldsymbol{\theta})$. We aim to estimate parameters $\boldsymbol{\theta}$ following the principles of maximum likelihood estimation.

1.2.2 Observed cancer incidence

We focus on cancer diagnoses observed within our defined study period and target population. As described in Section 2.3 in main paper, we first categorize our data. We define a one-year date interval δ as the period from January 1st to December 31st of year δ. Similarly, we define a one-year age interval α spanning from age α up to, but not including, $\alpha + 1$ (measured in years). Additionally, we introduce the vectors $\boldsymbol{\delta}$ and $\boldsymbol{\alpha}$ to represent the sets of these date and age intervals, respectively.

Next, we define an indicator variable $\mathcal{I}_{\delta,\alpha,i}$ which equals 1 if subject i is diagnosed with cancer in cell (δ,α), and 0 otherwise:

$$\mathcal{I}_{\delta,\alpha,i} = I(D_i \in \delta) \cdot I(A_i \in \alpha) \cdot I(A_i < C_i) \tag{1}$$

For example, $\mathcal{I}_{2010,55,i} = 1$ indicates that subject i was diagnosed with cancer in year 2010 at age 55.

We also define $N_{\delta,\alpha,x}$ as the *observed* total number of cancer cases in cell (δ, α) among participants in group $X = x$, for $x \in \{0, 1\}$. Formally,

$$N_{\delta,\alpha,x} = \sum_{i=1}^{n_x} \mathcal{I}_{\delta,\alpha,i}$$

For completeness we also define the total number of cancer cases in each group as:

$$N_{\bullet,\bullet,x} = \sum_{\delta\in\boldsymbol{\delta}} \sum_{\alpha\in\boldsymbol{\alpha}} N_{\delta,\alpha,x}$$

Let $n_{\delta,\alpha,x}$ denote the person-years at risk (i.e., those without cancer who could develop it) in cell (δ, α) in group $X = x$. In practice, these numbers can be approximated by the count of individuals still alive in each cell, which is usually available from official statistics. Observed cancer incidence in year δ and age group α for participants in group x (denoted as $\lambda_{\delta,\alpha,x}$) is estimated as

$$\widehat{\lambda}_{\delta,\alpha,x} = \frac{N_{\delta,\alpha,x}}{n_{\delta,\alpha,x}}$$

1.2.3 Counterfactual cancer incidence

First, for each cancer case i invited to the programme ($X_i = 1$), we derive the probability of being diagnosed in cell (δ, α) in world 0 (as if they were not invited) as a function of the parameters $\boldsymbol{\theta}$. For symptomatic cancers invited to the programme, this probability equals 1 for the cell in which cancer is diagnosed and 0 for all other cells, as their lead time is assumed to be zero. Formally, we write this probability as:

$$P\Big(\mathcal{I}^{(0)}_{\delta,\alpha,i} = 1 \mid X_i = 1, M_i = symp, D_i = d, A_i = a\Big) = I(d \in \delta) \cdot I(a \in \alpha) \tag{2}$$

Consistent with the definition in equation (1), $\mathcal{I}^{(0)}_{\delta,\alpha,i}$ is defined as

$$\mathcal{I}^{(0)}_{\delta,\alpha,i} = I(D^{(0)}_i \in \delta) \cdot I(A^{(0)}_i \in \alpha) \cdot I(A^{(0)}_i < C^{(0)}_i)$$

Calculating the probability that a screen-detected case i invited to the programme would be diagnosed with cancer in cell (δ, α) in world 0 is less trivial and depends on the parameters $\boldsymbol{\theta}$. Supplementary Figure S1 illustrates our approach to computing this probability. Consider the i-th subject, whose cancer was detected based on screening test on April 19, 2016 at age 67.8 years (black dot in Supplementary Figure S1a). We want to calculate their probability of being diagnosed with cancer in world 0 in each cell. First, note that any cell not lying on the subject's life trajectory is assigned a probability of zero (grey zeros in Supplementary Figure S1a). In contrast, the probability that the subject i is diagnosed with cancer in any other cell depends on both the lead time distribution (parameterized by $\boldsymbol{\theta}$; dotted black line in Supplementary Figure S1b) and on whether the subject remains alive and under study at that time (the yellow, green and red line in Supplementary Figure S1b). To calculate the probability that subject i would be diagnosed with cancer in world 0 at a specific cell - for example, at ($\delta = 2017$, $\alpha = 68$), labeled as p_3 in Supplementary Figure S1a - one integrates the relevant portion (blue area) of the lead time distribution multiplied by the probability that the subject remains alive and under observation (black solid line in Supplementary Figure S1b).

Formally, we write this probability as:

$$\begin{aligned} &P\Big(\mathcal{I}^{(0)}_{\delta,\alpha,i} = 1 \mid X_i = 1, M_i = scr, D_i = d, A_i = a, \boldsymbol{U_i} = \boldsymbol{u}, \boldsymbol{\theta}\Big) \\ &= \int_0^\infty I\big((d + l) \in \delta\big) \cdot I\big((a + l) \in \alpha\big) \cdot P(a + l < C_i \mid a < C_i, \boldsymbol{u}) \cdot f_L(l \mid \boldsymbol{\theta})dl \\ &= \int_{l_{\text{start}}}^{l_{\text{end}}} P(a + l < C_i \mid a < C_i, \boldsymbol{u}) \cdot f_L(l \mid \boldsymbol{\theta})dl \end{aligned} \tag{3}$$

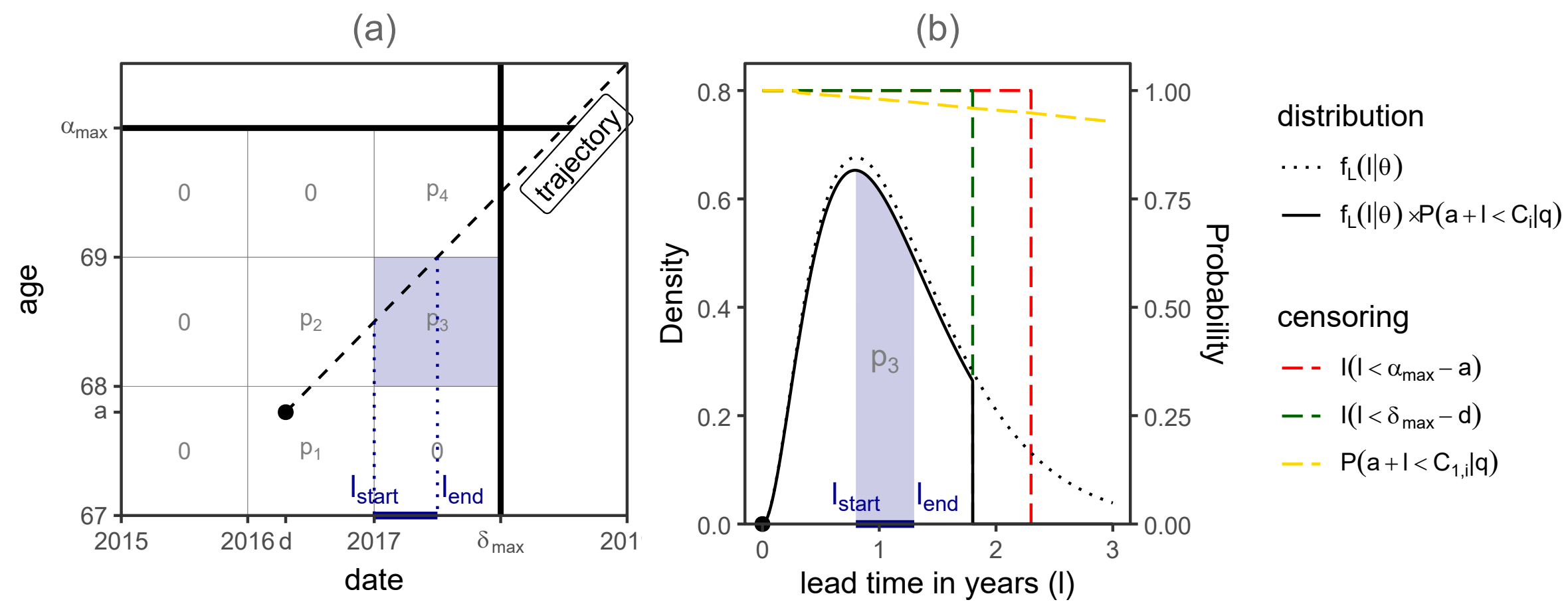

Figure S1: Illustration of probability calculation for the i-th subject using equation 3. (a) The black dot corresponds to the cancer diagnosis made based on a screening test for subject i. The trajectory (dashed line) illustrates how age for subject i changes over time. All future events for subject i must fall on this trajectory. (b) The dashed lines (red, green, and yellow) show the probability (secondary y axis) that subject i remains alive and under observation after lead time is added. The dotted black line represents the density function (primary y axis) of lead time, given parameters $\boldsymbol{\theta}$ (in this case assumed to take certain values). The solid black line is obtained by multiplying the probabilities by the lead time density function.

where

$$\begin{aligned} P(a+l<C_i \mid a<C_i,\boldsymbol{u}) =& P(a+l<C_{1,i} \mid a<C_{1,i},\boldsymbol{u}) \cdot I(a+l<C_{2,i}) \cdot I(a+l<C_{3,i}) \\ =& P(a+l<C_{1,i} \mid a<C_{1,i},\boldsymbol{u}) \cdot I(l<\delta_{max}-d) \cdot I(l<\alpha_{max}-a) \end{aligned}$$

and $\boldsymbol{U_i}$ denotes the vector of covariates for subject i used to estimate non-cancer mortality (e.g. sex, date of birth, date at cancer diagnosis). The term $P(a+l<C_{1,i} \mid a<C_{1,i},\boldsymbol{u})$ represents the probability (survival function) that subject i remains alive (i.e., does not die from other causes) after the lead time l is added. This probability can be obtained using population mortality tables. The limits of integration in Equation 3 are equal to:

$$l_{\text{start}} = \max(\min(\delta)-d, \min(\alpha)-a)$$

$$l_{\text{end}} = \min(\min(\delta)-d, \min(\alpha)-a)+1$$

where $\min(\alpha)$ and $\min(\delta)$ denote the lower bounds of intervals α and δ, respectively. For δ, the lower bound corresponds to January 1st of year δ.

In shorthand, we write the probability that cancer i, invited to the programme, would be detected in world 0 in cell (δ,α) as follows:

$$p^{(0)}_{\delta,\alpha,i}(\boldsymbol{\theta}) = P\Big(\mathcal{I}^{(0)}_{\delta,\alpha,i}=1 \mid X_i=1, M_i, D_i=d, A_i=a, \boldsymbol{U_i}=\boldsymbol{u},\boldsymbol{\theta}\Big) =$$

$$= \begin{cases} I(d\in\delta)\cdot I(a\in\alpha) \text{ , if } M_i = symp, \\ \int_{l_{start}}^{l_{end}} P(a+l<C_i \mid a<C_i,\boldsymbol{u}) \cdot f_L(l \mid \boldsymbol{\theta})dl \text{ , if } M_i = scr, \\ 0 \text{ , if } M_i = noc \end{cases}$$

and the probability that *cancer* i is excess is equal to $1-\sum_{\delta\in\boldsymbol{\delta}}\sum_{\alpha\in\boldsymbol{\alpha}} p^{(0)}_{\delta,\alpha,i}(\boldsymbol{\theta})$.

The counterfactual cancer incidence in each cell can be estimated as:

$$\widehat{\lambda}^{(0)}_{\delta,\alpha}(\boldsymbol{\theta}) = \frac{\sum_{i=1}^{n_1} p^{(0)}_{\delta,\alpha,i}(\boldsymbol{\theta})}{n_{\delta,\alpha,1}}$$

where $\sum_{i=1}^{n_1} p^{(0)}_{\delta,\alpha,i}(\boldsymbol{\theta})$ is the expected number of cancer cases in world 0 in cell (δ, α).

1.2.4 Likelihood

Our goal is to find parameters $\boldsymbol{\theta}$ that minimizes the overall discrepancy (across all cells) between $\widehat{\lambda}_{\delta,\alpha,0}$ and $\widehat{\lambda}^{(0)}_{\delta,\alpha}(\boldsymbol{\theta})$. Several methods could be used to achieve this; here, we adopt a maximum likelihood approach.

We derive the likelihood in several steps. To simplify the derivation, we impose two assumptions that can be relaxed later: (a) the data arise from a randomized controlled trial launched at the start of the screening programme, with one arm invited and the other not; and (b) both arms recruit an equal number of participants each year (i.e. person-years at risk is the same between the two groups across all cells). In the first step, we write the likelihood function in a similar manner as we would write the likelihood for the multinomial distribution, linking probabilities (fixed part) to observed counts (random part). First, we focus solely on individuals who were not invited to the programme and express the likelihood as follows:

$$L_1(\boldsymbol{\theta}) = \prod_{i=1}^{n_0} (1-w)^{I(M_i^{(1)} \in \{noc\})} \quad (4)$$
$$(w \cdot p_\Delta(\boldsymbol{\theta}))^{I(M_i^{(0)} \in \{noc\} \,\cap\, M_i^{(1)} \in \{scr\})}$$
$$\prod_{\delta \in \boldsymbol{\delta}} \prod_{\alpha \in \boldsymbol{\alpha}} (w \cdot p_{\delta,\alpha}(\boldsymbol{\theta}))^{I(\mathcal{I}^{(0)}_{\delta,\alpha,i}=1)}$$

where w denotes the probability of being a cancer cases in world 1, $p_\Delta(\boldsymbol{\theta})$ denotes the probability that a cancer case in world 1 is excess, and $p_{\delta,\alpha}(\boldsymbol{\theta})$ denotes the probability that a cancer case in world 1 would be diagnosed in world 0 within cell (δ, α). Formally:

$$w = P(A^{(1)} < C^{(1)})$$

$$p_{\delta,\alpha}(\boldsymbol{\theta}) = E_{M,D,A,\boldsymbol{U}|X=1,M\in\{scr,symp\},\boldsymbol{\theta}}\left(p^{(0)}_{\delta,\alpha,i}(\boldsymbol{\theta})\right)$$

$$p_\Delta(\boldsymbol{\theta}) = 1 - \sum_{\delta \in \boldsymbol{\delta}} \sum_{\alpha \in \boldsymbol{\alpha}} p_{\delta,\alpha}(\boldsymbol{\theta})$$

The likelihood (4) is entirely hypothetical, since it assumes that each subject can be observed in both worlds. We can understand the individual likelihood as follows. If an individual is not diagnosed with cancer in world 1 (and, by assumptions, also in world 0), then their contribution is the probability of not being diagnosed with cancer in world 1; if an individual is diagnosed with cancer by screening in world 1 but is not diagnosed with cancer in world 0 then their contribution to the likelihood is the probability of being an excess case (which is a function of parameters $\boldsymbol{\theta}$); lastly, if an individual is diagnosed with cancer in world 0 in a cell (δ, α) then their contribution to the likelihood is the probability of being diagnosed with cancer in world 0 in that cell given parameters $\boldsymbol{\theta}$.

In the second step, we simplify the likelihood L_1 by removing any terms that do not depend on the parameters being estimated ($\boldsymbol{\theta}$):

$$L_2(\boldsymbol{\theta}) = \prod_{i=1}^{n_0} p_\Delta(\boldsymbol{\theta})^{I(M_i^{(0)} \in \{noc\} \,\cap\, M_i^{(1)} \in \{scr\})} \prod_{\delta \in \boldsymbol{\delta}} \prod_{\alpha \in \boldsymbol{\alpha}} p_{\delta,\alpha}(\boldsymbol{\theta})^{I(\mathcal{I}^{(0)}_{\delta,\alpha,i}=1)} =$$
$$= p_\Delta(\boldsymbol{\theta})^{N_{\Delta,0}} \cdot \prod_{\delta \in \boldsymbol{\delta}} \prod_{\alpha \in \boldsymbol{\alpha}} p_{\delta,\alpha}(\boldsymbol{\theta})^{N^{(0)}_{\delta,\alpha,0}} \quad (5)$$

where $N_{\Delta,0}$ is the number of excess cases we would observe in world 1 among subjects not invited to the programme. This quantity can be calculated as the difference in cancer count between world 1 and world 0:

$$N_{\Delta,0} = N^{(1)}_{\bullet,\bullet,0} - N^{(0)}_{\bullet,\bullet,0}$$

Note that L_2 is proportional to L_1. In the last step, we replace the probabilities as well as the number of excess cancer cases with their plug-in estimates to obtain the final likelihood:

$$L_3(\boldsymbol{\theta}) = \widehat{p}_\Delta(\boldsymbol{\theta})^{\widehat{N}_{\Delta,0}} \cdot \prod_{\delta\in\boldsymbol{\delta}} \prod_{\alpha\in\boldsymbol{\alpha}} \widehat{p}_{\delta,\alpha}(\boldsymbol{\theta})^{N_{\delta,\alpha,0}} \tag{6}$$

where

$$\widehat{p}_{\delta,\alpha}(\boldsymbol{\theta}) = \frac{1}{N_{\cdot,\cdot,1}} \sum_{i=1}^{n_1} p^{(0)}_{\delta,\alpha,i}(\boldsymbol{\theta}) \cdot I(M_i \in \{scr, symp\})$$

$$\widehat{p}_\Delta(\boldsymbol{\theta}) = 1 - \sum_{\delta\in\boldsymbol{\delta}} \sum_{\alpha\in\boldsymbol{\alpha}} \widehat{p}_{\delta,\alpha}(\boldsymbol{\theta})$$

Since we assume that equal number of cancer cases are recruited in each group, $N_{\Delta,0}$ can be estimated simply as a difference in cancer count between invited and non-invited group:

$$\widehat{N}_{\Delta,0} = N_{\cdot,\cdot,1} - N_{\cdot,\cdot,0}$$

The parameters $\boldsymbol{\theta}$ are estimated by maximizing the likelihood L_3. Note that compared to the likelihood L_2, the likelihood L_3 cannot be formulated at the individual level because we cannot identify which observations are excess cases. Instead, the total number of excess cases is being replaced by their estimates.

1.2.5 Relaxing assumptions

We propose a simple and robust correction to account for differences in person-years at risk between the invited and non-invited groups. These differences may vary across cells (δ, α), with the invited group having more person-years at risk in some cells and the non-invited group in others.

We define $n^*_{\delta,\alpha} = \min(n_{\delta,\alpha,0}, n_{\delta,\alpha,1})$ as the target sample size in each cell. Our correction proceeds by downweighting both the probabilities and associated observations in the likelihood L_3 (equation (7)). First, we adjust the observations:

$$N^*_{\delta,\alpha,x} = \widehat{\lambda}_{\delta,\alpha,x} \cdot n^*_{\delta,\alpha}$$

$$\widehat{N}^*_{\Delta,0} = \sum_{\delta\in\boldsymbol{\delta}} \sum_{\alpha\in\boldsymbol{\alpha}} \left(N^*_{\delta,\alpha,1} - N^*_{\delta,\alpha,0}\right)$$

The weighted data $N^*_{\delta,\alpha,0}$, $N^*_{\delta,\alpha,1}$ can be interpreted as the adjusted number of cancer cases in the invited and non-invited groups if both groups were of size $n^*_{\delta,\alpha}$.

We also adjust the probabilities $\widehat{p}_{\delta,\alpha}(\boldsymbol{\theta})$ by downweighting each patient's contribution to the estimated probabilities using the appropriate weights (denoted by w^*_i):

$$\widehat{p}^*_{\delta,\alpha}(\boldsymbol{\theta}) = \frac{1}{N^*_{\cdot,\cdot,1}} \sum_{i=1}^{n_1} p^{(0)}_{\delta,\alpha,i}(\boldsymbol{\theta}) \cdot I(M_i \in \{scr, symp\}) \cdot w^*_i$$

$$\widehat{p}^*_\Delta(\boldsymbol{\theta}) = 1 - \sum_{\delta\in\boldsymbol{\delta}} \sum_{\alpha\in\boldsymbol{\alpha}} \widehat{p}^*_{\delta,\alpha}(\boldsymbol{\theta})$$

where $N^*_{\cdot,\cdot,1}$ is the number of cancer cases in the invited group and the weight w^*_i is defined as:

$$w^*_i = \sum_{\delta\in\boldsymbol{\delta}} \sum_{\alpha\in\boldsymbol{\alpha}} I(X_i = 1 \cap A_i < C_i) \cdot I(D_i \in \delta \cap A_i \in \alpha) \cdot \frac{n^*_{\delta,\alpha}}{n_{\delta,\alpha,1}}$$

The likelihood takes the following form:

$$L_4(\boldsymbol{\theta}) = \widehat{p}^*_\Delta(\boldsymbol{\theta})^{\widehat{N}^*_{\Delta,0}} \cdot \prod_{\delta\in\boldsymbol{\delta}} \prod_{\alpha\in\boldsymbol{\alpha}} \widehat{p}^*_{\delta,\alpha}(\boldsymbol{\theta})^{N^*_{\delta,\alpha,0}} \tag{7}$$

The variance of the MOCCI estimator can be further reduced by modelling the incidence in the non-invited group using an age-period-cohort model. This approach is applied in the empirical example (see Supplementary Section 3).

1.3 Estimating the probability of overdiagnosis

We estimate the probability of overdiagnosis using a competing risks framework. Specifically, for each screen-detected cancer in world 1, we ask what would have happened had they not been invited to the screening programme (in world 0). Two mutually exclusive events are considered: symptomatic detection of the cancer (that would occur in world 0) and overdiagnosis. For subject i observed in world 1 let T_i be the time from the (screen) diagnosis to the first event in world 0, and $\epsilon_i \in \{\text{symp, O}\}$ the corresponding cause indicator (symptomatic detection vs. overdiagnosis). We then define the cause-specific absolute risks up to time t by:

$$\begin{aligned} AR_{symp,i}(t \mid \boldsymbol{\theta}) =& P(T_i < t, \epsilon_i = \text{symp} \mid X_i = 1, M_i = scr, A_i = a, \boldsymbol{U_i} = \boldsymbol{u}, \boldsymbol{\theta}) \\ =& \int_0^t S_{C_{1,i}}(a + s \mid \boldsymbol{u}) \cdot f_L(s \mid \boldsymbol{\theta}) ds \end{aligned}$$

$$\begin{aligned} AR_{O,i}(t \mid \boldsymbol{\theta}) =& P(T_i < t, \epsilon_i = \text{O} \mid X_i = 1, M_i = scr, A_i = a, \boldsymbol{U_i} = \boldsymbol{u}, \boldsymbol{\theta}) \\ =& \int_0^t P(s < L \mid \boldsymbol{\theta}) \cdot f_{C_{1,i}}(a + s \mid \boldsymbol{u}) ds = \\ =& \int_0^t P(s < L \mid \boldsymbol{\theta}) \cdot \lambda_{C_{1,i}}(a + s \mid \boldsymbol{u}) \cdot S_{C_{1,i}}(a + s \mid \boldsymbol{u}) ds \end{aligned}$$

Here, $f_{C_{1,i}}$, $\lambda_{C_{1,i}}$, and $S_{C_{1,i}}$ are the density function, hazard function and the survival function for event C_1 for subject i and can be obtained from population mortality tables. The probability that person i is overdiagnosed in her lifetime given parameters $\boldsymbol{\theta}$ can be calculated as:

$$P(O_i = 1 \mid X_i = 1, M_i = scr, A_i = a, \boldsymbol{U_i} = \boldsymbol{u}, \boldsymbol{\theta}) = \lim_{t \to \infty} AR_{O,i}(t \mid \boldsymbol{\theta})$$

Absolute risks and the probability of overdiagnosis can be estimated for each individual by substituting the true parameter values $\boldsymbol{\theta}$ with their maximum likelihood estimates $\widehat{\boldsymbol{\theta}}$ obtained from equation (7). We obtain the marginal (population) absolute risk for each cause by taking the expectation of the individual absolute risk with respect to A and $\boldsymbol{U}$:

$$AR_{symp}(t \mid \boldsymbol{\theta}) = E_{A,\boldsymbol{U} \mid X=1, M=scr, \boldsymbol{\theta}}(AR_{symp,i}(t \mid \boldsymbol{\theta}))$$

$$AR_O(t \mid \boldsymbol{\theta}) = E_{A,\boldsymbol{U} \mid X=1, M=scr, \boldsymbol{\theta}}(AR_{O,i}(t \mid \boldsymbol{\theta}))$$

$$P(O = 1 \mid X_i = 1, M_i = scr, \boldsymbol{\theta}) = \lim_{t \to \infty} AR_O(t \mid \boldsymbol{\theta})$$

In practice, we estimate the theoretical expectation by the empirical average over the sample. In the empirical example we illustrate that the proportion of overdiagnosed cases can be decomposed into two components: one for progressive cancers and another for non-progressive cancers.

Section 2: Simulation study

2.1 Aims

The first aim of this simulation study is to illustrate that the MOCCI method satisfies all key large-sample properties of a maximum-likelihood estimator (consistency, asymptotic normality) in the simplest scenario, where all cancers are progressive, lead time follows an exponential distribution, and only one parameter needs to be estimated. The second aim is to evaluate whether the method can accurately estimate the parameters in a more complex setting where both the parameters of the lead time distribution and the proportion of non-progressive cancers must be jointly estimated. Specifically, we want to examine whether the method returns a value close to zero when the true proportion of non-progressive cancers is zero, and a non-zero value when the true proportion is non-zero.

2.2 Setup

2.2.1 Data generating mechanism (DGM)

This simulation study is based on empirical data from the Slovenian breast cancer screening programme, where screening is done every two years. We selected data on cancer cases invited to the programme aged between 50 and 75 years, from 2008 to 2020. Approximately one third of patients were symptomatically detected. From this dataset, we drew two independent bootstrap samples to construct our simulated data: Sample 0 and Sample 1. Both samples were of equal size, with sample sizes $\{2000, 4000, 8000\}$ each. Sample 1 was kept unchanged, as it corresponds to the invited group ($X = 1$). On the other hand, Sample 0 corresponds to the non-invited group ($X = 0$), therefore lead time was added to each screen-detected case to simulate their date and age at symptomatic detection (symptomatic cancers were left unchanged). Lead time, measured in years, was drawn from a mixture distribution. Each screen-detected case had probability π of receiving infinite lead time, representing non-progressive cancers. With probability $(1 - \pi)$, lead times were generated from an exponential distribution with rate parameter λ. We generated data under two DGMs: DGM-A ($\lambda = 0.5$, $\pi = 0$) and DGM-B ($\lambda = 0.5$, $\pi = 0.2$). The lead time distribution in each scenario is plotted in Supplementary Figure S2. To increase the speed of the simulation, all patients were assumed to remain alive in the period between screen-detection and symptomatic detection. Cases for which the simulated symptomatic detection occurred outside the predefined study period (2008-2020) or target population (50-75 years) are excluded from Sample 0 (so-called excess cases). The two datasets, Sample 0 and Sample 1, form our input data. The flowchart depicting the data generating mechanism is shown in Supplementary Figure S3.

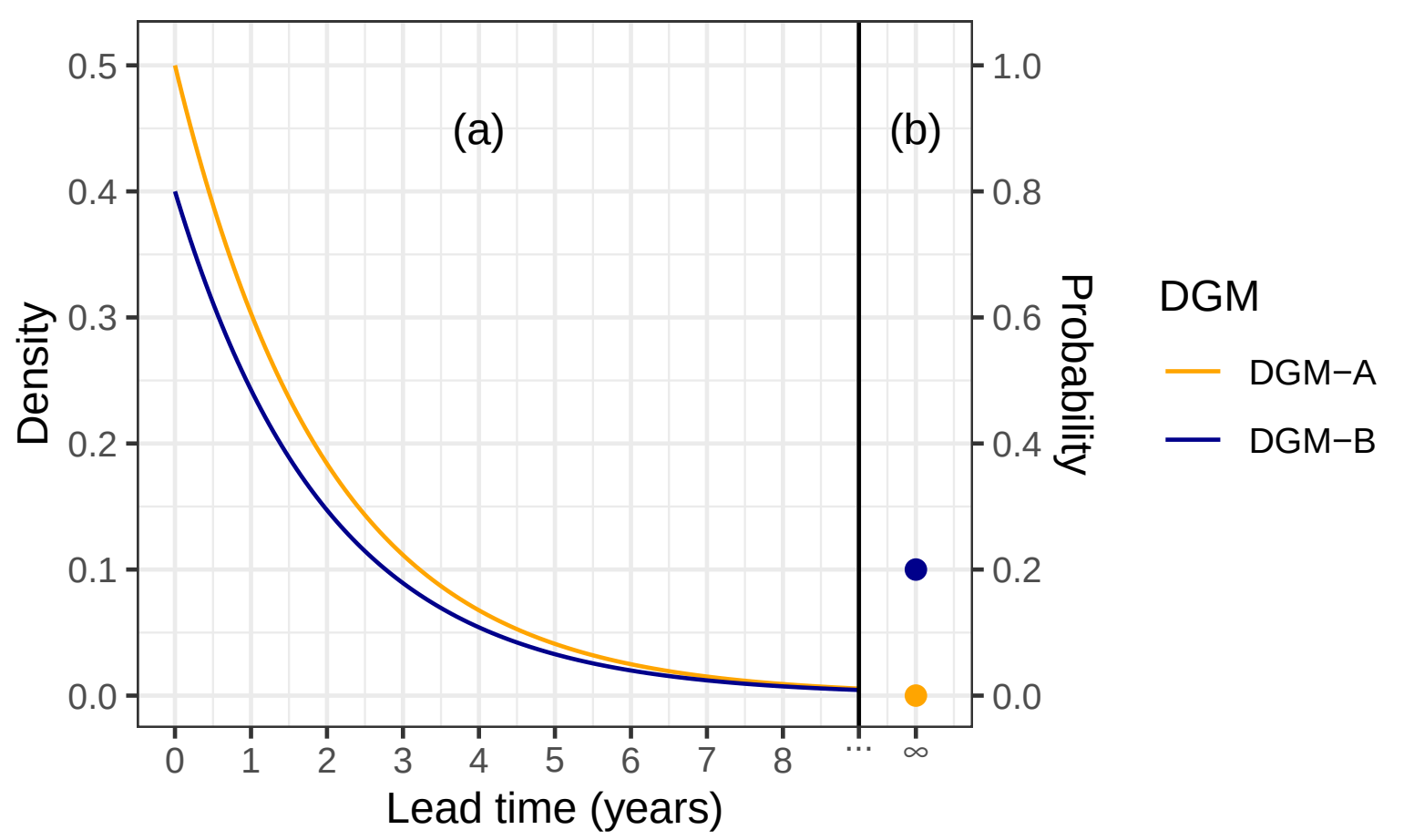

Figure S2: Lead time distribution for screen-detected cancers under DGM-A ($\lambda = 0.5$, $\pi = 0$) and DGM-B ($\lambda = 0.5$, $\pi = 0.2$). (a) The density of progressive cancers for exponential distribution with rate λ (primary y-axis). (b) The proportion (π) of non-progressive cancers, where $L \to \infty$ (secondary y-axis).

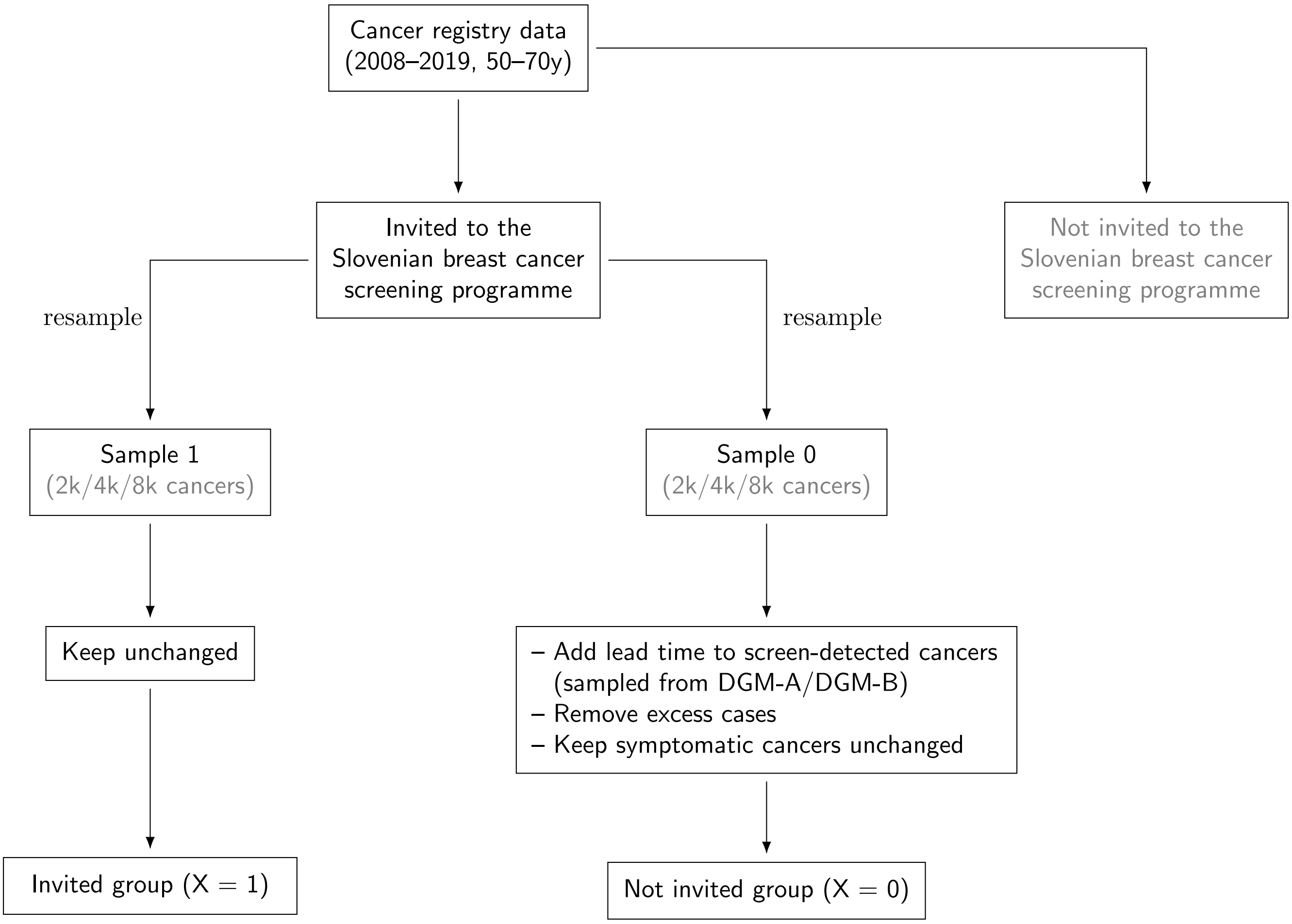

Figure S3: Flowchart of the data generating mechanism used in the simulation study.

2.2.2 Estimands

The estimands of interest are the parameters of lead time distribution, λ and π (proportion of non-progressive cancers).

2.2.3 Methods

We used the unweighted MOCCI method to estimate the parameters of the lead time distribution. Two models of lead time were fitted to the data: model A, with density $f_A(\lambda)$, which corresponds to the exponential model used to generate lead time in DGM-A; and model B, with density $f_B(\lambda, \pi)$, which corresponds to the mixture model used to generate lead time in DGM-B. Essentially, model A is a special case of model B with π constrained to 0.

2.2.4 Performance measures

First, to examine whether MOCCI retains the desirable MLE properties, we generated 1000 DGM-A data sets at each sample size and, in every replicate, fitted model A to the data and estimated the parameter λ. The usual MLE performance metrics were then summarised (Section 2.3.1). Second, to evaluate how well the method recovers lead time parameters when model B is fitted to the data, we simulated the data 1000 times under both DGM-A and DGM-B for each sample size. For each replicate we fitted model B and reported the mean (and variance) of $\widehat{\lambda}$ and $\widehat{\pi}$. These values were subsequently compared with their corresponding true parameter values (Section 2.3.2).

2.2.5 Hardware, software and computing time

Computations were performed on a 64-bit Windows system with a 12th Gen Intel Core i9-12900K processor (3.20 GHz, 16 cores) and 32 GB RAM, using R version 4.2.2. When model A was fitted to the data, the computing time of the MOCCI method was approximately 5, 7, and 10 minutes for sample sizes of 2000, 4000, and 8000, respectively. These times increased to 9, 13, and 16 minutes when model B was fitted to the data. All reported computing times refer to single-core execution.

2.3 Results

2.3.1 Properties of the MOCCI method

First, we assessed whether the MOCCI method possesses the desirable properties of the MLE in the simplest setting, with data generated under DGM-A and model A fitted to the data. The only parameter being estimated in this case was λ. The performance metrics of the MLE, summarized in Supplementary Table S1, indicate that the MOCCI method is a consistent estimator that is asymptotically normally distributed. It also exhibits the expected root-n consistency: the ratios $\sqrt{\mathrm{var}(\widehat{\lambda}_{4000})}/\sqrt{\mathrm{var}(\widehat{\lambda}_{2000})} = 0.76$ and $\sqrt{\mathrm{var}(\widehat{\lambda}_{8000})}/\sqrt{\mathrm{var}(\widehat{\lambda}_{4000})} = 0.68$ are both close to the theoretical value $1/\sqrt{2} \approx 0.707$.

Table S1: Performance of the MOCCI method (DGM-A, model A)

**Property**	**Performance measure for $\widehat{\lambda}$**	**Target**	**Empirical value (n = 2000, 4000, 8000)**
Consistency	Mean	0.5	0.501, 0.501, 0.501
	Standard deviation	$\to 0$	0.025, 0.019, 0.013
	RMSE	$\to 0$	0.025, 0.019, 0.013
Asymptotic normality	Shapiro–Wilk p-value	> 0.05	0.154, 0.396, 0.616
Variance ratio (empirical / model)	$\dfrac{\mathrm{Var}(\widehat{\lambda})}{\widehat{\mathrm{Var}}(\widehat{\lambda})}$	1	0.95, 1.01, 0.92

2.3.2 Accuracy in the presence of non-progressive cancers

Secondly, we examined how well the method performs in a more difficult situation when in addition to λ we also wish to estimate the proportion of non-progressive cancers (π). The main challenge in this case is that different combinations of λ and π can yield similar likelihood values (i.e. the two parameters are correlated). This arises because the observed excess in cancer incidence can be explained either by a long (but finite) lead time (captured by parameter λ) or by allowing some cancers to have infinite lead time values (captured by parameter π). This correlation persists even when $\pi = 0$.

The simulation results are shown in Supplementary Table S2. When the true proportion of non-progressive cancers is 0 (DGM-A) or 0.2 (DGM-B), the rate of convergence is slower and the variance is much higher than in the single-parameter setting of Supplementary Table S1. This is the consequence of added complexity in untangling two correlated parameters (rare but long lead times and non-progressive incidence) from the same data.

Table S2: Parameter estimates for Model B fitted using the MOCCI method to data generated under DGM-A and DGM-B

DGM	Model		Mean estimates (standard deviation)		
			n = 2000	n = 4000	n = 8000
DGM-A ($\lambda = 0.5, \pi = 0$)	$f_B(\lambda, \pi)$	λ	0.51 (0.035)	0.51 (0.026)	0.51 (0.021)
		π	0.00 (0.010)	0.00 (0.008)	0.00 (0.008)
DGM-B ($\lambda = 0.5, \pi = 0.2$)	$f_B(\lambda, \pi)$	λ	0.46 (0.073)	0.48 (0.060)	0.49 (0.043)
		π	0.16 (0.040)	0.18 (0.029)	0.19 (0.020)

Section 3: Empirical example

3.1 Person-years at risk calculations

Cancer cases were straightforward to obtain from the national cancer registry and to link to the screening database via unique identifiers, so that each case could be assigned unambiguously to the invited or non-invited group. In contrast, constructing the corresponding person-years at risk for each group required additional work. Data from the Statistical office of the republic of Slovenia provided the numbers of women at risk at the beginning of each calendar year by age group. We converted these counts into person-years for each age–year cell using the method of Carstensen [1], which assumes constant mortality within each Lexis triangle. Person-years at risk for the invited group were then obtained from the Slovenian breast cancer screening programme database, which includes all invited women (with and without cancer). A key complication is that the programme does not invite women with a prior diagnosis of breast cancer. Because our incidence rates are intended to refer to the total population, and to make the invited and non-invited groups comparable, we applied a robust correction to account for this exclusion. As the denominators in the incidence calculations are large (population size) and the proportion of women with a previous cancer is small, the choice among reasonable correction methods has only a negligible impact on the results.

The correction was implemented cell-wise over all (δ, α) combinations; schematic illustration is presented in Supplementary Figure S4. For each cell (δ, α):

1. Using data from the cancer registry and the Statistical Office, and restricting to the period before programme implementation, we estimated for each age group α the proportion of women who had ever been diagnosed with breast cancer and were still alive. This was done under the assumption of stable cancer incidence. For cell (δ, α), this proportion was multiplied by the total population at risk in that cell to approximate the number of women who had ever been diagnosed with breast cancer and were still alive (at risk); this estimate is denoted by $n^{prev}_{\delta,\alpha}$.
2. Among women without a prior cancer in cell (δ, α) (i.e. the pool from which the programme actually invited women), we calculated the proportion of women who were invited to screening, denoted by $p^{inv}_{\delta,\alpha}$.
3. We then estimated how many women with a prior cancer should have been invited in cell (δ, α) if they had been eligible, denoted by $n^{add}_{\delta,\alpha}$, as $n^{add}_{\delta,\alpha} = n^{prev}_{\delta,\alpha} \cdot p^{inv}_{\delta,\alpha}$ (see Supplementary Figure S4).
4. We resampled $n^{add}_{\delta,\alpha}$ individuals (rounded to the whole nearest number) from the set of women actually invited in cell (δ, α) and added these resampled records to the screening database, thereby imputing a corresponding invited subgroup of women with prior cancer in that cell.
5. Using this augmented screening dataset we recalculated person-years at risk for each (δ, α) cell in the invited group.

Finally, person-years at risk for the non-invited group in each cell were obtained by subtracting the (corrected) invited person-years from the total person-years at risk.

3.2 Complete workflow

We applied the weighted MOCCI method to estimate the lead time distribution, which we modelled parametrically as

$$L \sim \begin{cases} \infty, & \text{with probability } \pi, \\ \mathrm{Exp}(\lambda_{\mathrm{exp}}), & \text{with probability } 1-\pi, \end{cases} \tag{8}$$

In model A, π was set to 0. Our aim was to estimate parameters $\boldsymbol{\theta} = \{\lambda_{exp}\}$ in model A and $\boldsymbol{\theta} = \{\lambda_{exp}, \pi\}$ in model B. The likelihood function used for estimation is given by (see Supplementary Section 1.2.5).

$$L_4(\boldsymbol{\theta}) = \widehat{p}^*_{\Delta}(\boldsymbol{\theta})^{\widehat{N}^*_{\Delta,0}} \cdot \prod_{\delta \in \boldsymbol{\delta}} \prod_{\alpha \in \boldsymbol{\alpha}} \widehat{p}^*_{\delta,\alpha}(\boldsymbol{\theta})^{N^*_{\delta,\alpha,0}} \tag{9}$$

The likelihood requires the following variables as inputs:

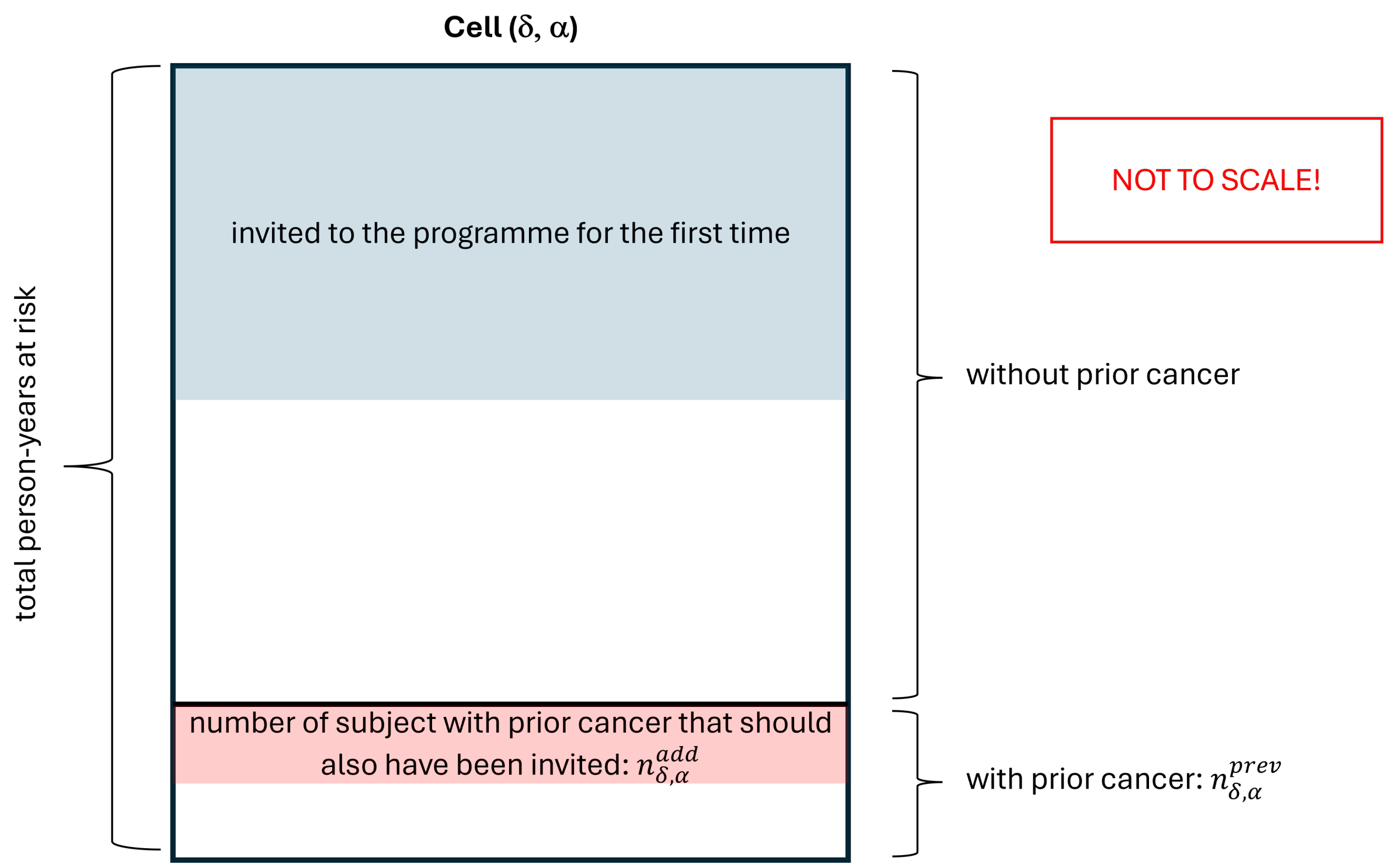

Figure S4: Schematic illustration of person-year calculations in the invited group (see text). Block areas represent relative magnitudes but are not to scale; in particular, the area corresponding to women with prior cancer is greatly exaggerated.

- The cancer count stratified by year and age among those not invited to the programme ($N^*_{\delta,\alpha,0}$).
- The difference in cancer count between the invited and not invited group ($\widehat{N}^*_{\Delta,0} = N^*_{\delta,\alpha,1} - N^*_{\delta,\alpha,0}$)
- The individual-level data for all cancers detected in the invited group, which are used to estimate $\widehat{p}^*_{\delta,\alpha}(\boldsymbol{\theta})$ and $\widehat{p}^*_{\Delta}(\boldsymbol{\theta})$

For each cell (δ, α), we set the target sample size equal to that of the invited group, i.e. $n^*_{\delta,\alpha} = n_{\delta,\alpha,1}$. Consequently, $\widehat{p}^*_{\delta,\alpha}(\boldsymbol{\theta})$, $\widehat{p}^*_{\Delta}(\boldsymbol{\theta})$ and $N^*_{\delta,\alpha,1}$ could be estimated directly from the data on the invited group without requiring any adjustments. The only quantity that needed adjustment was the cancer count in the non-invited group ($N^*_{\delta,\alpha,0}$).

The $N^*_{\delta,\alpha,0}$ was calculated in two steps. In the first step, we fitted an age–period–cohort model to the non-invited group in order to obtain cell specific baseline incidence rates $\widehat{\lambda}_{\delta,\alpha,0}$. We fitted a generalized additive age–cohort (APC-type) model with age and cohort as predictors and log person-years as an offset. Both age and cohort effects were modelled using penalised P-splines; smoothing parameters were selected by REML. The period effect was assumed to be negligible. In the second step, the estimated incidence rates $\widehat{\lambda}_{\delta,\alpha,0}$ were multiplied by $n^*_{\delta,\alpha}$ to obtain adjusted cancer counts for the non-invited group. The use of an age-period-cohort model not only enabled us to make predictions outside training data (during years 2019-2020) but also provided more stable estimates.

The two parameters, π and λ_{exp}, were estimated by numerically maximizing the likelihood L_4. The covariance matrix for these estimates was estimated using the observed Fisher information matrix. Because the two parameters (π and λ_{exp}) are correlated, we used Monte Carlo simulation from their joint normal distribution to obtain 95 % confidence intervals (CI) for derived quantities such as the lead time distribution, the AR_{symp} and AR_O (see Supplementary Section 1.3). Since the effective sample size of the non-invited group is much larger than the sample size used in the likelihood, the resulting confidence intervals are likely conservative.

References

[1] B. Carstensen. Age–period–cohort models for the lexis diagram. Statistics in medicine, 26(15):3018–3045, 2007.

[2] E. Goetghebeur, S. Le Cessie, B. De Stavola, E. E. Moodie, I. Waernbaum, and "on behalf of" the topic group Causal Inference (TG7) of the STRATOS initiative. Formulating causal questions and principled statistical answers. Statistics in Medicine, 39(30):4922–4948, Dec. 2020.

[3] M. A. Hernán and J. M. Robins. Causal Inference: What If. Boca Raton: Chapman & Hall/CRC., Oct. 2020.
